# Mother and Offspring in Conflict: Why Not?

**DOI:** 10.1371/journal.pbio.1002084

**Published:** 2015-03-18

**Authors:** Francisco Úbeda, Andy Gardner

**Affiliations:** 1 School of Biological Sciences, Royal Holloway University of London, Egham, United Kingdom; 2 School of Biology, University of St Andrews, St Andrews, United Kingdom; Institute of Science and Technology Austria (IST Austria), AUSTRIA

## Abstract

A gene mediating interactions between mouse mothers and their pups has recently been claimed to support coadaptation rather than the kinship theory of genomic imprinting. This Formal Comment argues that this claim is unfounded.

## Introduction

Genomic imprinting (GI) is the differential expression of genes according to whether they have been inherited from an individual’s mother or father [1]. An imprinted gene may be expressed when maternally inherited and silenced when paternally inherited (“maternally expressed”) or expressed when paternally inherited and silenced when maternally inherited (“paternally expressed”) [1]. A number of theories have been proposed to explain this evolutionary puzzle [2].

The “kinship theory” suggests that GI is borne out of a conflict between maternally inherited versus paternally inherited genes [3]. Specifically, if an individual is more related to its social partners through its mother than its father, selection favours reduced expression of genes for selfishness and increased expression of genes for selflessness when those genes are inherited from its mother and increased expression of genes for selfishness and reduced expression of genes for selflessness when those genes are inherited from its father [3]. The outcome is silencing of maternally inherited genes for selfishness and paternally inherited genes for selflessness [3]. The reverse pattern is expected if relatedness is greater through the individual’s father [3].

The “coadaptation theory” instead suggests that GI enables coordination between mother and offspring [4]. Specifically, if an offspring achieves greater fitness when its gene products match those of its mother, this may favour silencing of the offspring’s paternally inherited genes, as these are less likely to be carried and expressed by its mother [4]. Conversely, if an offspring achieves greater fitness when its gene products differ from those of its mother, this may favour silencing of the offspring’s maternally inherited genes, as these are more likely to be carried and expressed by its mother [5].

**Fig 1:**
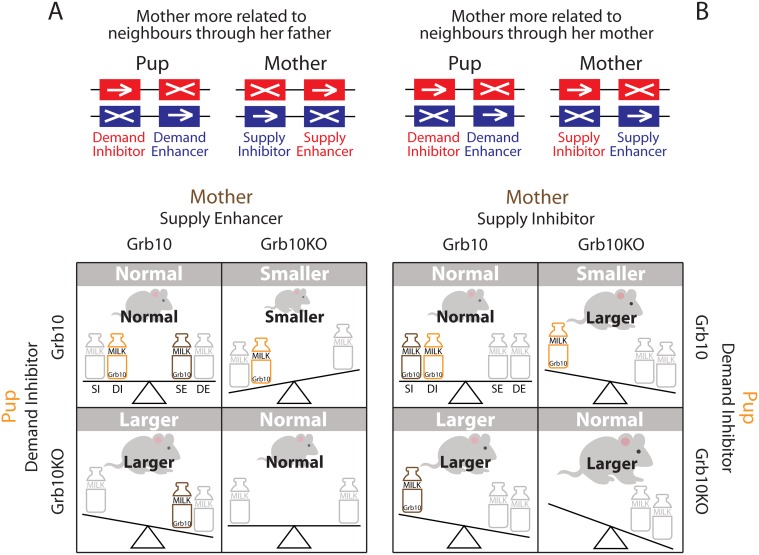
Experimental results and theoretical predictions. Red and blue colourings correspond to genes of maternal and paternal origin, respectively. Orange and brown colourings correspond to genes expressed in the pup and the mother, respectively. Gray colouring corresponds to genes contributing to the phenotype but not considered in the current experiment. Arrowed and crossed-out rectangles correspond to expressed and silenced genes, respectively. All possible pairings between the wild type *Grb10* and the loss-of-function mutant *Grb10KO* in the pup and mother are presented in a 2 × 2 matrix. In each cell, we indicate (i) the experimental result in white over grey background and (ii) the theoretical prediction in black. The amount of milk a pup obtains results from a tension between offspring demand and maternal supply. Eliminating genes that maintain this tension (by rendering them nonfunctional) alters the amount of milk the pup obtains. (A) If the mother is more related to the recipients of allo-maternal care through her father, then in the pup, demand-inhibitor (DI) genes should be paternally silenced and demand-enhancer (DE) genes should be maternally silenced; in the mother, supply-inhibitor (SI) genes should be maternally silenced and supply-enhancer (SE) genes should be paternally silenced; and so, as *Grb10* is maternally expressed in pup and mother, this gene is predicted to be a DI in the pup and a SE in the mother, and the corresponding predicted 2 × 2 matrix of pup weights exactly matches that observed in the experiment. (B) If the mother is more related to the recipients of allo-maternal care through her mother, then in the pup, DI genes should be paternally silenced and DE genes should be maternally silenced; in the mother, SI genes should be paternally silenced and SE genes should be maternally silenced; and so, as *Grb10* is maternally expressed in pup and mother, this gene is predicted to be a DI in the pup and a SI in the mother, and the corresponding predicted 2 × 2 matrix of pup weights only partially matches that observed in the experiment. Accordingly, the kinship theory suggests that, in the natural setting in which GI has evolved, the mother is more related to the recipients of her allo-maternal care through her father than through her mother.

Cowley et al. [6] report a study of mouse-pup weight and lean-to-fat mass ratio mediated by *Grb10*, which is maternally expressed in the pup and in the mother’s mammary tissue. In particular, they describe the pup’s weight when the pup and/or the mother have their maternally inherited copies of *Grb10* knocked out, which leads to (1) a large pup when the pup—but not the mother—carries the knockout, (2) a small pup when the mother—but not the pup—carries the knockout, and (3) a normal-weight pup when the pup and mother both carry the knockout (Fig. 1) [6,7]. Cowley et al. [6] suggest that these data are not readily explained by the kinship theory and are instead supportive of the coadaptation theory. Here, we show that the kinship theory is fully compatible with the data and that the coadaptation theory struggles to explain the observations of Cowley et al. [6].

## The Kinship Theory Can Explain the Experimental Data

The kinship theory was initially explored in the context of embryos and newborns [8]. An individual who is more demanding of its mother reduces the availability of resources for its maternal siblings and, since these are not always paternal siblings, natural selection favours silencing of maternally inherited genes underpinning the extraction of maternal resources (demand enhancers) and silencing of paternally inherited genes underpinning the preservation of maternal resources (demand inhibitors) [9–10]. Accordingly, the kinship theory readily explains why *Grb10* is imprinted in the pup: it is more related to its maternal siblings through its mother than through its father. The predicted direction of imprint depends on *Grb10*’s function when expressed in pups: it is predicted to be paternally expressed if it is a milk-demand enhancer, and maternally expressed if it is a milk-demand inhibitor (Fig. 1) [9–10].

Moreover, the kinship theory also explains GI in mothers, in relation to the tradeoff between maternal care of their own pup versus allo-maternal care of other females’ pups (contra Cowley et al. [6]) [11–13]. If a mother is more related to the recipients of her allo-maternal care through her father than through her mother, natural selection favours silencing of genes promoting maternal care (supply enhancers) when paternally inherited and genes promoting allo-maternal care (supply inhibitors) when maternally inherited [11–13]. And the reverse pattern is expected if she is more related to them through her mother than her father [11–13]. Accordingly, the kinship theory readily explains why *Grb10* is imprinted in mouse mothers. The predicted direction of imprint depends on *Grb10*’s function when expressed in the mother: a milk-supply enhancer will be maternally expressed when relatedness is greater through her father but paternally expressed when relatedness is greater through her mother, and the reverse will be true for a milk-supply inhibitor (Fig. 1).

Finally, the kinship theory makes specific predictions about the phenotypes resulting from the interaction of mutant mothers and pups (contra Cowley et al. [6]). Given that it is maternally expressed, *Grb10* is a supply enhancer if the mother is more related to the recipients of her allo-maternal care through her father, and a supply inhibitor if she is more related to them through her mother. In the first scenario, this leads to a predicted 2 × 2 matrix of pup weights that exactly matches that observed by Cowley et al. (Fig. 1, panel A) [6]. In the second scenario, a different 2 × 2 matrix is obtained (Fig. 1. panel B). Accordingly, the kinship theory narrows the set of all possible 2 × 2 matrices to two very specific alternatives, one of which perfectly matches the experimental data. And it yields a further testable prediction: that a mouse mother is generally more related to the recipients of her allo-maternal care through her father than through her mother.

## The Coadaptation Theory Struggles to Explain the Experimental Data

The coadaptation theory predicts GI in offspring. Specifically, it predicts maternal expression if matching of gene products increases offspring fitness [4], and it predicts paternal expression if matching of gene products decreases offspring fitness [5]. The observed maternal expression of *Grb10* in mouse pups is consistent with the former rather than the latter scenario.

However, the coadaptation theory does not predict GI in adults and struggles to explain the observed imprinting of *Grb10* in mouse mothers. This is because the mother’s maternally inherited and paternally inherited copies of *Grb10* are equally likely to be carried and expressed in the offspring, so the silencing of either copy would not promote a match. Cowley et al. [6] suggest that imprinting in mouse mothers may be nonadaptive and merely represents the imprinting of pups lingering into adulthood.

Moreover, Cowley et al.’s [6] pup-weight data do not clearly support the coadaptation theory. First of all, the complementarity of gene functions between mother and pup is a feature of both cooperative and conflicted interactions (as shown above), and thus such complementarity per se does not support the coadaptation theory. Secondly, the coadaptation theory requires that pups gain fitness by matching their gene products with those of their mothers [4], and, for this to be consistent with the observed data, larger pups would need to be less fit than average-sized pups. Cowley et al. [6] have not shown this, and, indeed, it may be more likely that pup fitness increases with pup weight [14–15], at least within the normal range of allelic variation within which coadaptation-mediated GI has allegedly evolved.

## Conclusions

Cowley et al. [6] have suggested that their data are supportive of the coadaptation theory and do not support the kinship theory. Here, we have suggested that the exact opposite is true. Whilst the kinship theory explains imprinting of *Grb10* in pups and mothers [11–13,16], the coadaptation theory can only explain imprinting in the pups. And whilst the kinship theory makes direct predictions about pup weight that are supported by the data if the population structure results in greater relatedness through the father, the coadaptation theory instead makes predictions about pup fitness that are not supported by the data.

This disagreement stems, in part, from a conceptual misunderstanding about the adaptive agents involved in this evolutionary conflict. Cowley et al. [6] describe the conflict as occurring between mother and father and hence are unable to see how the kinship theory could explain imprinting in the mother’s mammary tissue, as this contains only the mother’s genes. However, the conflict is really occurring between maternally inherited and paternally inherited genes, and, as an individual’s mammary tissue contains genes inherited from her father as well as her mother, it too represents a battleground for intragenomic conflict.

## References

[1] Reik W, Walter J. (2001) Genomic imprinting: parental influence on the genome. Nat. Rev. 2:21–32 10.1038/35047554 11253064

[2] Spencer HG, Clark AG. (2014) Non-conflict theories for the evolution of genomic imprinting. Heredity 113: 112–118 10.1038/hdy.2013.129 24398886 PMC4105448

[3] Haig D. (1997) Parental antagonism, relatedness asymmetries, and genomic imprinting. Proc. R. Soc. Lond. B 264: 1657–1662 9404029 10.1098/rspb.1997.0230 PMC1688715

[4] Wolf JB, Hager R. (2006) A maternal-offspring coadaptation theory for the evolution of genomic imprinting. PLoS Biol 4:e380 10.1371/journal.pbio.0040380 17105351 PMC1635750

[5] Patten MM, Ross L, Curley JP, Queller DC, Bonduriansky R, Wolf JB. (2014) The evolution of genomic imprinting: theories, predictions, and empirical tests. Heredity 113: 119–128 10.1038/hdy.2014.29 24755983 PMC4105453

[6] Cowley M, Garfield AS, Madon M, Charalambous M, Clarkson RW et al. (2014) Developmental programming mediated by complementary roles of imprinted Grb10 in mother and pup. PLoS Biol 12: e1001799 10.1371/journal.pbio.1001799 24586114 PMC3934836

[7] Wilkins JF. (2014) Genomic imprinting of the Grb10: coadatation or conflict? PLoS Biol 12: e1001800 10.1371/journal.pbio.1001800 24586115 PMC3934815

[8] Moore T, Haig D. (1991) Genomic imprinting in mammalian development: a parentl tug-of-war. Trends Genetics 7: 45–49. 2035190 10.1016/0168-9525(91)90230-N

[9] Haig D. (1996). Placental hormones, genomic imprinting, and maternal fetal communication. J. Evol. Biol. 9: 357–380.

[10] Úbeda F, Haig D. (2003) Dividing the child. Genetica 117: 103–110 12656577 10.1023/a:1022320801661

[11] Úbeda F, Gardner A. (2010) A model for genomic imprinting in the social brain: juveniles. Evolution 64: 2587–2600 10.1111/j.1558-5646.2010.01015.x 20394663

[12] Úbeda F, Gardner A. (2011) A model for genomic imprinting in the social brain: adults. Evolution 65: 462–475 10.1111/j.1558-5646.2010.01115.x 20812976

[13] Úbeda F, Gardner A. (2012) A model for genomic imprinting in the social brain: elders. Evolution 66: 1567–1581 10.1111/j.1558-5646.2011.01517.x 22519791

[14] Krackow S. (1993) The effect of weaning weight on offspring fitness in wild house mouse (*Mus musculus domesticus*): a preliminary study. Ethology 95: 76–82

[15] Krackow S. (1997) Maternal investment, sex-differential prospects, and the sex ratio in wild house mice. Behav. Ecol. Sociobiol. 41: 435–443

[16] Wilkins JF, Haig D. (2003) Inbreeding, maternal care and genomic imprinting. J. Theor. Biol. 221:559–564. 12713940 10.1006/jtbi.2003.3206

